# Epidemiology of suicidal ideation, suicide attempts, and direct self-injurious behavior in adolescents with a migration background: a representative study

**DOI:** 10.1186/s12887-019-1404-z

**Published:** 2019-02-01

**Authors:** Carolin Donath, Marie Christine Bergmann, Sören Kliem, Thomas Hillemacher, Dirk Baier

**Affiliations:** 1 Center for Health Services Research in Medicine, Department of Psychiatry and Psychotherapy, Friedrich-Alexander-Universität Erlangen-Nürnberg, Schwabachanlage 6, 91054 Erlangen, Germany; 2 Criminological Research Institute of Lower Saxony, Lützerodestr. 9, 30161 Hannover, Germany; 3 Center for Addiction Research, Clinic for Psychiatry, Social Psychiatry and Psychotherapy, Hannover Medical School, Carl-Neuberg-Str. 1, 30625 Hannover, Germany; 4 Department of Psychiatry and Psychotherapy, Paracelsus Medical University Nuremberg, Prof.-Ernst-Nathan-Str. 1, 90419 Nürnberg, Germany; 5 Institute of Delinquency and Crime Prevention, ZHAW School of Social Work, Pfingstweidstrasse 96, Postfach, 8037 Zurich, Switzerland

**Keywords:** Adolescent, Cross-cultural comparison, Suicidal ideation, Suicide, attempted/statistics & numerical data, Self-injurious behavior/epidemiology, Cross-sectional studies, Human migration, Pediatrics/epidemiology

## Abstract

**Background:**

Data on the prevalence of suicidal ideation, suicide attempts, and direct self-injurious behavior in adolescents with a migration background are scarce. There are hints that this population is at risk. The aim of the study is to investigate the epidemiology of suicidal ideation, suicide attempts, and direct self-injurious behavior in adolescents with a migration background in Germany while taking gender-specific differences into consideration.

**Methods:**

A representative study with *N* = 10,638 students (mean age 14.91 years, SD = .73).) in the state of Lower Saxony in Germany was conducted. In the 2014–2015 school year, 672 classes were selected by randomly sampling different school types. The participation rate was 84.1%, excluding any classes for which the director refused to provide consent. A total of 49.8% were female adolescents, and 23.3% of the participants had a migration background. Target variables were assessed with items from the Ottawa Self-Injury Inventory, the Self-Harm Behavior Questionnaire and the Self-Harm Inventory, partly adapted.

**Results:**

Of all students, 7.6% had a lifetime history of suicide attempts, and 36.6% answered with a rating of at least “rarely” when asked to rate the lifetime prevalence of suicidal ideation. The 12-month prevalence of direct self-injurious behavior was 17.8%. Adolescents with a migration background showed a significantly higher prevalence of all three constructs (*p* = .006; *p* < .001; p = .006). Male students with a migration background reported a significantly higher lifetime prevalence of suicide attempts (4.7% vs. 3.1%) than native males (*p* = .009). Female students with a migration background reported a significantly higher lifetime prevalence of suicide attempts (15.9% vs. 10.4%) and suicidal ideation (“often” 12.1% vs. 8.9%) than native female students (p < .001; *p* = .008).

**Conclusion:**

Our assessment indicates an elevated risk for suicidal behaviors in adolescents with a migration background. From research on adults, it is known that the dominant motives for suicidal behavior in migrants are associated with their migration history/situation. As suggested by Cramer and Kapusta’s (Front Psychol 8:1756, 2017) theoretical model, the Social-Ecological Framework of Theory, Assessment, and Prevention, there is a need for culturally sensitive preventions that take into account the specific reasons for suicide attempts in migrants.

**Electronic supplementary material:**

The online version of this article (10.1186/s12887-019-1404-z) contains supplementary material, which is available to authorized users.

## Background

Suicidal ideation and non-suicidal self-injury are relatively frequent conditions in adolescents [2–4]. For a certain percentage of these adolescents, these suicidal ideations cross the intention-behavior gap [5, 6] into suicide attempts.

According to the World Health Organization, one of the most important risk factors for suicide is a previous suicide attempt [7]. It is known that some vulnerable groups such as people with a migration background who might experience discrimination have a higher risk for suicide [7], and this is reflected in the rate of suicide attempts. Therefore, research on the epidemiology of suicidal ideation and suicide attempts in vulnerable groups such as migrants is important and necessary for coming up with preventive measures. Furthermore, there are data that show the association of non-suicidal self-injury and suicide attempts [8]. Prior or current non-suicidal self-injury counts as a risk factor for suicide [1]. Thus, knowledge about non-suicidal self-injurious behavior in vulnerable groups is also required. To date, no representative data for adolescents with a migration background are available for Germany concerning suicidal ideation, suicide attempts, and direct self-injurious behavior.

### What is known?

First, we present an overview of the three concepts covered in this manuscript and what is known about their epidemiology in adolescence.

#### Non-suicidal self-injury / deliberate self-harm: Definition and epidemiological data in adolescents internationally and in Germany

In the literature, deliberate self-harm is often used interchangeably with the term “non-suicidal self-injury” (NSSI) and describes the intentional injuring of a person’s own body without suicidal intentions [9]. However, in some instances the term “self-harm” includes also self-harming actions irrespective of the extent of suicidal intent, thus including possible suicidal intentions [10]. Pattison & Kahan’s very early definition was further developed and specified until NSSI was included in the DSM-V when the functional, emotional, and motivational aspects of NSSI were taken into consideration [11]. The lifetime prevalence of suicidal harm is reported to be 25.6% in German adolescents [2], and the 12-month prevalence of NSSI is 19.8% [2]. Another (representative) German study reported a rate of 14.9% as the 12-month prevalence of NSSI [12]. A systematic review including 52 international studies that analyzed the prevalence of NSSI in adolescents reported a rate of 18% (lifetime prevalence), which was not significantly different from the prevalence reported in the studies that used the alternative term “deliberate self-harm” [4]. In the study here it was aimed to investigate the epidemiology of self-injurious behavior, in the sense of the definition used by Brunner et al. [13]: intentional self-inflicted damage to the surface of an individual’s body regardless of the suicidal intent, which is labeled direct self-injurious behavior by the authors. Thus, in the following the term direct self-injurious behavior will be used.

#### Suicidal ideation: Definition and epidemiological data in adolescents internationally and in Germany

Suicidal ideation is defined as “thoughts of engaging in behavior intended to end one’s life” [14]. Brunner et al. [12] reported a rate of 14.4% of suicidal ideation (no more specification stated) in a representative sample of German students. In a gender-specific analysis in this study [15], rates of 19.8% for female individuals and 9.3% for male individuals were reported. Another representative study in Germany reported rates of 39.4% for a lifetime prevalence of suicidal ideation (at least “rarely”); the lifetime prevalence rate of having suicidal ideations at least “sometimes” was 15.6% [16]. The European-wide ESPAD study, which included 17 countries, reported the prevalence of only “thoughts of self-harm,” but the study did not make clear whether suicidal ideation or NSSI was assessed (“Have you ever thought of harming yourself?”). The rate of having had such thoughts at least 5 times varies from 2.1 to 15.3% (median 7.4%) in European countries [3]. In a representative study of Mexican adolescents, the lifetime prevalence of suicidal ideation was 11.5% [17]. A systematic review of US data on suicidal ideation in adolescents reported rates between 19.8 and 24.0% for the lifetime prevalence and between 15.0 and 29.0% for the 12-month prevalence [14]. The review also stated the range of rates from cross-national WHO studies on suicidal ideation in adolescents: a world-wide lifetime prevalence of 21.7 to 37.9% and a 12-month prevalence of 11.7 to 26.0%.

#### Suicide attempts: Definition and epidemiological data in adolescents internationally and in Germany

Suicide attempts are defined as “engagement in potentially self-injurious behavior with at least some intent to die” [14]. The distinction from NSSI (“without intention to die”) is obvious. For Germany, studies have reported a lifetime history of suicide attempts in adolescents of 6.5% [2], 8% (10.9% female adolescents, 4.9% male adolescents) [12, 15], and 9.0% [16]. In the international context, the median rate was 10.5% pooled across 17 European countries, whereas the European data ranged from 4.1 to 23.5% [3], which emphasizes clinically relevant differences between countries. Representative data for adolescents in Mexico indicated a lifetime prevalence of 3.1%. A review of US-based data claimed a lifetime prevalence between 3.1 and 8.8% and a 12-month prevalence between 7.3 and 10.6% [14]. A review of the cross-national WHO studies identified rates between 1.5 and 12.1% for the lifetime prevalence and between 1.8 and 8.4% for the 12-month prevalence pooled across 28 countries in different continents [14].

#### What is known about adolescents with a migration background?

The data on the epidemiology of suicidal ideation, suicide attempts, and also on self-harm/NSSI in adolescents with a migration background are still scarce. Due to increases in world-wide migration rates and increases in the numbers of refugees, it is urgently necessary to know about the mental health of those growing groups in (Western) societies in order to modify prevention measures for culture sensitivity. This goes along with the Social-Ecological Suicide Prevention Model (SESPM) [1], which suggests that studies need to take into account macro-level conditions such as the new society’s cultural conditions, norms, and values to which a migrant has to adapt and eventually acculturate.

According to Esser, who developed a theoretical framework on migration aspects and processes [18–20], there can be be distinguished four different facets describing the extent in which an individual is included in the a) society of origin and b) in the society of the country where it immigrated to. If an individual is neither integrated in any system respectively society, one speaks of marginality. There is multiple inclusion, if the integration has taken place for both – the originating as well as the majority society; if they individual remains to be integrated in the origin society only it is called individual segmentation or segregation and at last if the individual has completely adapted to the majority society and has given up the social integration in the originating society it is called individual assimilation. Esser differentiates between the processes of acculturation, integration and assimilation. Assimilation as an extent in the state of similarity to the majority society can have cultural, structural, social and identificative aspects. In an empirical analysis Esser [21] showed that the multiple inclusion is not disadvantageous in comparison to assimilation concerning social and structural aspects of integration, however unfavorable outcomes were seen for ethnic segmentation. The framework of Esser is based on the fourfold model of acculturation of John Berry [22]. This results also in four facets of acculturation depending on a) how valuable it is for an individual to maintain relationships in the larger society and b) how valuable it is to maintain the identity and characteristics of the origin society. Thus, four combination possibilities result: integration (both are valuable), assimilation (the relationships in the larger society where the individual immigrated to are most important), segregation (only maintaining the characteristics and values of the origin society is seen as valuable) and marginalization (neither relationships in the larger society nor the identity of the origin society are valued as important).

Possibly, differences in the states of integration and assimilation between adolescents and their families could lead to underlying conflicts that can foster in vulnerable developmental phases like puberty psychic problems or suicidal ideations. There is the possibility of arising problems in the time of findings one’s own identity if the adolescent himself feels more assimilated – socially and identificative – than the older generation of his family who might expect a stronger identification with the culture of origin. There are hints that for certain risky health behaviors high assimilation of adolescents with migration background was a risk factor, while attitudes that favored segregation and a stronger attachment of the parents to the country of origin was a protective factor [23].

Merbach, Wittig and Brähler found not only a higher symptom load for anxiety and depression in migrants but also showed an association of the symptoms with the extent of assimilation and sensed discrimination [24].

A recent German study found that adolescents with a migration background showed a higher lifetime prevalence of both NSSI and suicide attempts [25]: 17.9% of adolescents with a migration background had a history of suicide attempts, but only 3.2% of their native counterparts did. Furthermore, 42.9% of adolescents with a migration background reported deliberate self-harm, but only 19.2% of “German” adolescents did. However, the study was not representative and included a rather small sample of *N* = 452 adolescents.

The results of a recent representative Swiss study of > 7000 participants [26] were less clear: For suicide attempts, the lifetime prevalence varied from 5.0% (Swiss Natives) to 7.3% (first-generation immigrants), whereas the 12-month prevalence for suicidal ideation ranged from 25.9% (Swiss Natives) to 31.0% (second-generation immigrants). However, a regression analysis revealed a lower risk for suicidal ideation when migration background was evident. Migration background was also not associated with suicide attempts.

A pooled analysis of European data stemming from the WHO/EURO study showed that the pattern of an increase in the lifetime prevalence of suicide attempts existed in adults with a migration background (15+ years) in comparison with the native population in 27 of the 56 immigrant groups that were analyzed. Only in a minority of the groups that were analyzed did the persons with a migration background have a lower prevalence of suicidal behavior [27]. However, whether the findings from these data can be transferred to adolescents is questionable.

Because of the scarce European data and the lack of representative data on this question for adolescents in Germany, we carried out a representative study to explore the epidemiology of suicidal ideations, −attempts and direct self-injurious behavior in adolescents with a migration background. Our goal was to investigate whether young migrants would be found to be at higher risk for suicide attempts and thus might be found to be, as suggested by the WHO, an especially vulnerable group that is threatened by suicide.

### Aims

In this study, we aimed to investigate the epidemiology of direct self-injurious behavior, suicidal ideation, and suicide attempts in a representative sample of German adolescents, focusing on the specifics of adolescents with a migration background.

### Research questions

1. What are the frequencies of direct self-injurious behavior, suicidal ideation, and suicide attempts in adolescents with a migration background in comparison with adolescents without a migration background?
2. What are the gender differences in the epidemiological data for direct self-injurious behavior, suicidal ideation, and suicide attempts in adolescents with a migration background?

## Methods

### Design

The following analyses were based on a representative cross-sectional survey of ninth graders in the German state of Lower Saxony conducted in the spring of 2015 [28]. Every tenth German citizen lives in Lower Saxony, a German federal state (about eight million inhabitants). It represents the German average, e.g., regarding the economic situation (unemployment rate or the number of migrants).

The classes were randomly selected from all classes during the 2014–2015 school year. The aim was to survey one out of every eight classes. Since the classes vary in size from one school type to another, random sampling was done within various types of schools. The only school type not represented in the survey was that of special schools for students with disabilities.

**Fig. 1:**
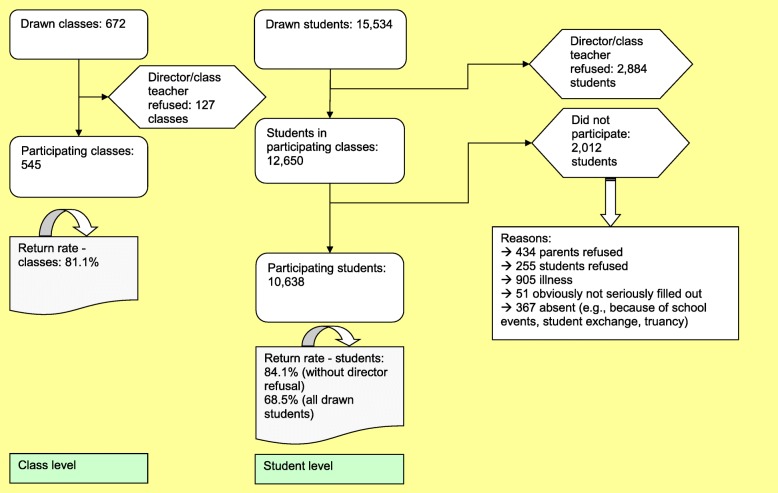
Sample composition

A total of 672 classes were selected for the survey. Because some school directors or teachers declined to participate, the survey was administered to a total of 545 classes where 12,650 students were enrolled, of which 10,638 students participated in the survey (see Fig. 1). The reasons for non-participation included illness (*n* = 905), missing parental consent (*n* = 434), refusal (*n* = 255), irrelevant questionnaires (*n* = 51), and other reasons (*n* = 367; e.g., school events, student exchange, truancy). The response rate was 84.1% for students who had the opportunity to participate because their director had agreed.

The survey was a self-reported criminological study and focused mainly on the assessment of delinquent behavior. A written survey was administered in classrooms by trained interviewers. They briefly described the survey to the class and were available to answer questions during the whole 90 min that respondents were given to complete the survey. Each student completed the questionnaire by him- or herself.

This study was approved by the state’s educational authority. The parents of the respondents received information about the study in advance with a request that they provide written consent for their child to participate. The students were also allowed to refuse to participate in the survey, regardless of their parents’ consent. The students were first reminded that the survey was voluntary, that there would be no negative consequences for refusing to participate, and that they had the right to refuse to answer any of the questions.

### Sample/participants

The mean age of the sample of 10,638 students was 14.91 years (SD = .73). 49.8% were female adolescents. A total of 23.3% were defined as having a migration background. 69.7% of all participating students reported living with both corporal parents, and 79.4% reported living with at least one sibling. 32.3% of the sample reported living in rural areas (< 10,000 inhabitants), 18.9% in small towns (10,000 to < 20,000 inhabitants), one fourth (26.2%) in medium-size cities (20,000 to < 50,000 inhabitants), and 22.7% in urban municipalities (50,000 or more inhabitants). 41.2% of the students stated that they would attempt to earn a university entrance (high school) diploma, which requires 12 or 13 years of school education. A further 44.9% of the participants were aiming for a secondary modern school certificate (10 years), and 13.9% were attempting a secondary general school certificate (9 years).

The sample included students with a migration background from different countries or regions of origin. The ethno-specific proportion of the total sample was: 6.4% Former Soviet Union, 4.2% Turkey, 2.7% Poland, 1.7% Former Yugoslavia, 1.6% Southern Europe, 2.0% Northern/Western Europe, 1.9% predominantly Islamic countries, 1.0% Asia, and 1.8% other countries. The migration-specific breakdown of the sample description is depicted in Table 1. The matter concerned in the majority adolescents with migration background of at least the second generation (96% of the adolescents with migration background).

**Table 1:** Description of the sample according to migration background

	N (10,638)	% female	% living with both corporal parents	% family living on social welfare	% attempting university entrance diploma
German	8157	49.3	69.4	8.0	43.1
Former Soviet Union	683	53.2	77.2	14.7	32.4
Turkey	448	52.4	80.4	24.0	31.6
Poland	292	55.9	56.9	13.8	31.7
Former Yugoslavia	183	45.0	73.9	28.0	27.1
Southern Europe^a^	171	51.9	61.4	11.5	29.8
Northern/Western Europe^b^	211	50.8	62.2	8.8	39.7
Predominantly Islamic countries^c^	198	50.2	75.7	38.7	36.2
Asia^d^	103	45.4	66.9	18.2	54.3
Other countries^e^	191	51.0	58.1	16.1	52.1

^a^Portugal, Spain, Italy, Greece; ^b^ France, Benelux, Great Britain, Scandinavia; ^c^ Egypt, Afghanistan, Algeria, United Arabian Emirates, Gambia, Iraq, Iran, Jordanian, Kurdistan, Kuwait, Lebanon, Morocco, Pakistan, Palestine, Tunisia, Senegal, Somalia, Sudan, South-Sudan, Syria; ^d^ Bangladesh, China, India, Indonesia, Japan, South-Korea, Philippines, Singapore, Sri Lanka, Taiwan, Thailand, Vietnam; ^e^ USA, Brazil

The sample was drawn so that it would be representative of one state of Germany (Lower Saxony). We checked whether the final sample corresponded to the composition of the population that was examined with school types: The extent to which the sample was representative of the population was determined to be satisfactory; for example, the percentage of students in secondary general schools was 5.4% in the sample and 5.4% in the population in the 2014–2015 school year. This also took into account special needs schools for learning disabilities (2.7 and 2.7%). The highest deviation between sample and population existed for high schools (33.4% population and 31.5% sample). Weighting factors were calculated and applied to account for the slight deviations between the sample and the population to ensure that the results would be representative of the school type.

### Instruments

In addition to age and sex as demographic variables, we assessed the variable “migration background.” The definition and operationalization of migration background was used in accordance with population surveys from the German Census Bureau (“Mikrozensus”) carried out by the Federal Agency for Statistics (“Statistisches Bundesamt”) [29] and consisted of four variables: students’ place of birth, parents’ place of birth, and students’ and parents’ citizenship. A student was defined as having a migration background if at least one parent was born outside of Germany, if the student was born outside of Germany him- or herself or had a non-German citizenship, or if the student had at least one parent with a non-German citizenship.

The lifetime prevalence of suicidal ideations was assessed with the question: “Have you ever had suicidal thoughts?” with the four answer categories “no, never,” “yes, rarely,” “yes, sometimes,” and “yes, often.” The item has been used in other surveys before [16, 28] and was developed by the Criminological Research Institute of Lower Saxony. The wording is related to the Ottawa Self-Injury Inventory [30]; however, there were five answer categories instead of four, and there was a stronger time relation to the past year since it captured the 12-month prevalence rate instead of the lifetime prevalence rate.

The question “Have you ever seriously tried to commit suicide?” with the answer categories “yes” and “no” was used to assess the lifetime prevalence of suicide attempts. The item was also developed by the Criminological Research Institute of Lower Saxony and has been utilized and evaluated before [16, 28]. It corresponds to the assessment of suicide attempts in the Self-Harm Behavior Questionnaire [31] and to the wording in the Ottawa Self-Injury Inventory [30], taking into account the language differences in the expression of German and English.

The 12-month prevalence of direct self-injurious behavior was assessed with the item “In the last 12 months, did you intentionally cut, burn, carve, or injure yourself in another way?” with the answer categories “yes” and “no.” The item has been used before [28] and was developed by the Criminological Research Institute of Lower Saxony. The formulation of the item is a shortened version of the Self-Harm Inventory [32], it does not emphasize on the type of motive or the extent of suicidal intent.

### Statistical analysis

Data were analyzed with IBM SPSS Statistics 21. For the epidemiological analysis, sample data were weighted according to the population. Missing data in variables used for sample characterization (rates of missing data below 3%) was imputed by computing a regression of all other quantitative variables. The variable “migration background” and the dependent variables were imputed conservatively: If data were missing, the variable was imputed conservatively with “no” so that we would not erroneously raise the prevalence rate. The variable sex (rate of missing data 0.2%) was imputed after investigating the distribution of male and female individuals in the population of Lower Saxony, birth year 2000, in the German Census Bureau [33]. Analyses were conducted by applying descriptive and inference statistical methods. Chi^2^ tests were used to test for significant differences in frequencies. To account for problems with multiple testing (see, e.g., [34]) and the inflation of the Type I error rate, we used Bonferroni and Holm’s correction method [35, 36]. It is an extension of Bonferroni’s conservative correction formula [37] but offers simple, general, correct, and consistent advantageous over the original [38]. In this study, we applied nine significance tests. The p-levels of the tests were sorted in ascending order and compared with growing p-level barriers. The calculated barriers are: .005, .006, .007, .008, .010, .013, .017, .025, .050. The *p*-value of each individual test result has to be smaller than its corresponding barrier in order to be interpreted as significant. As a sensitivity analysis research question 1 was also computed with the second-generation-immigrant sample only in comparison to adolescents without migration background. The results are made available to the reader in Additional file 1. There, the operationalization of migration background was additionally to the above mentioned prerequisites amended with the item to be born in Germany.

## Results

We analyzed data from 10,638 adolescents. A total of 7.6% of all participants answered “yes” to the question of whether they had ever seriously attempted suicide. The 12-month prevalence for direct self-injurious behavior in the whole sample was 17.8%. The percentages of adolescents who reported having had suicidal ideation “often” was 5.9%, “sometimes” was 9.5%, and “rarely” was 20.8%; thus, 63.7% reported that they had never had suicidal thoughts. For completeness, the prevalence numbers based on un-imputed data: 7.8% for lifetime history of suicide attempt, 18.3% as 12-month prevalence for direct self-injurious behavior and 37.3% for lifetime prevalence of any suicidal thoughts [28].

### Research question 1: Epidemiology of suicidal ideation, suicide attempts, and direct self-injurious behavior in adolescents with a migration background

Adolescents with a migration background (*N* = 2481) reported a higher prevalence of all three investigated variables than adolescents without a migration background (*N* = 8157). These differences were statistically significant according to the corrected significance levels. While adolescents with a migration background reported a higher lifetime prevalence of suicide attempts (*p* < .001) and a higher 12-month prevalence of direct self-injurious behavior (*p* = .006), the lifetime prevalence of suicidal ideation differed explicitly in the category “yes, often” between adolescents with and without a migration background (p = .006) (Table 2).

**Table 2:** Frequency (in %) of suicidal ideation and suicide attempts (lifetime prevalence) and direct self-injurious behavior with regard to migration background

*N* = 10,638		No Migration background	Migration background	Chi^2^	p-value	Bonferroni-Holms corrected sign. Level
Suicidal ideation	No	64.1	62.6	12.538	**.006**	
	Yes, rarely	21.0	20.3			
	Yes, sometimes	9.5	9.7			
	Yes, often	5.5	7.4			.007
Suicide attempts		6.7	10.5	37.286	**<.001**	.005
Direct self-injurious behavior (12-month prevalence)		17.2	19.6	7.434	**.006**	.008

**Bold:** statistically significant

Un-imputed data:

*No Migration Background:* Suicidal ideation 62.8% (never), 21.7% (rarely), 9.8% (sometimes) 5.7% (often); Suicide attempts 6.9%; Direct self-injurious behavior 17.8%.

*Migration Background:* Suicidal ideation 61.8% (never), 20.8% (rarely), 9.9% (sometimes) 7.5% (often); Suicide attempts 10.7%; Direct self-injurious behavior 20.1%.

Reported prevalence of un-imputed data conforms to the numbers reported in final research report [28].

**Fig. 2:**
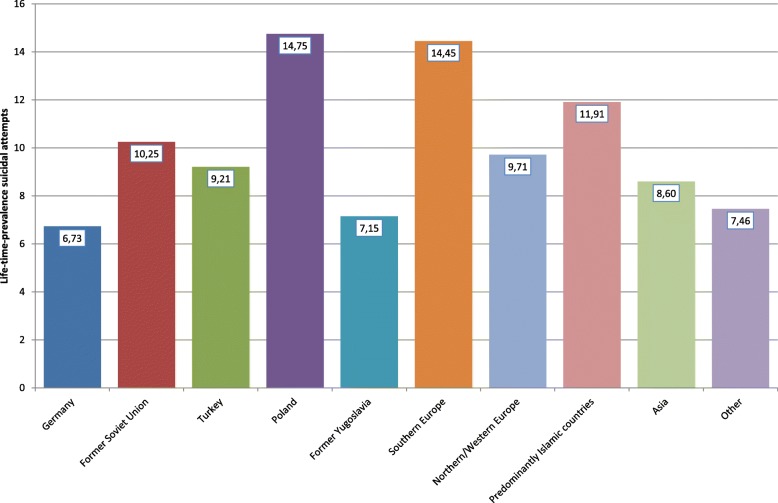
Lifetime prevalence of suicide attempts with regard to groups with different migration backgrounds

**Fig. 3:**
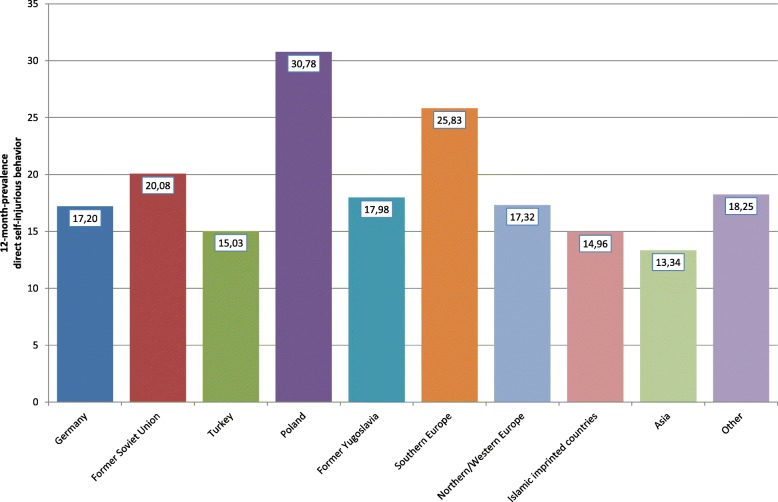
Twelve-month prevalence of direct self-injurious behavior with regard to groups with different migration backgrounds

**Fig. 4:**
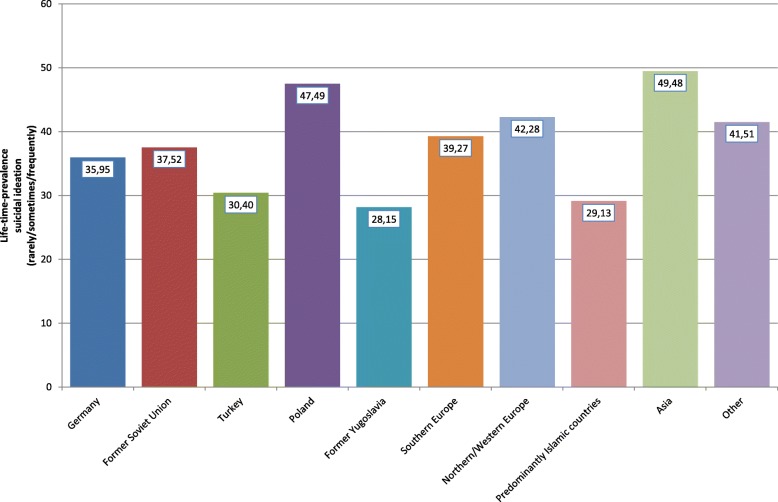
Lifetime prevalence of suicidal ideation with regard to groups with different migration backgrounds

Looking at the specific countries or regions of origin of the adolescents with a migration background, it can be seen that especially students with Polish or Southern European roots as well as students with roots in predominantly Islamic countries (not Turkey) affirmed a positive lifetime prevalence of suicide attempts (Fig. 2); whereas adolescents with a migration background from the former Yugoslavia reported almost the same prevalence as adolescents without a migration background. A similar but less clear picture could be seen with respect to the 12-month prevalence of direct self-injurious behavior: Students with roots in Poland or Southern Europe showed the highest prevalence, whereas adolescents from other regions (e.g., Asia, Turkey, or predominantly Islamic countries) even reported a lower 12-month prevalence of self-injuring in comparison with German adolescents without a migration background (Fig. 3). For suicidal ideation (i.e., intention without action), the ethno-specific results differed: While again students from Poland and Southern Europe as well as from North-/West Europe reported a higher lifetime prevalence of suicidal thoughts than adolescents without a migration background, the highest lifetime prevalence of suicidal ideation was reported by students with an Asian migration background (Fig. 4). The numbers presented for the prevalence of suicidal ideation are frequencies for the categories “rarely,” “sometimes,” and “often.”

### Sensitivity analysis

The results of Research Question 1 can be replicated when analyzing second generation immigrants only in comparison to adolescents without migration background. A significant higher rate of suicidal ideation and attempts are evident also in this subsample. The results are depicted in Additional file 1.

### Research question 2: Gender specifics in the epidemiology of suicidal ideation, suicide attempts, and direct self-injurious behavior in adolescents with a migration background

Female adolescents reported a higher prevalence of suicidal ideation, suicide attempts, and direct self-injurious behavior. This finding applies equally to adolescents with and without a migration background.

For example, female adolescents showed about a 3 times higher rate of suicide attempts in their case histories than male adolescents – independent of whether they had a migration background or not (Tables 3 and 4). However, in the gender-specific analysis, female adolescents with a migration background showed a significantly higher rate of suicide attempts than female adolescents without a migration background (*p* < .001). This finding also held for male adolescents with a migration background who had a significantly higher lifetime prevalence of suicide attempts than male adolescents without a migration background (*p* = .009). For both genders, the rate was about 1.5 times higher for individuals with a migration background.

**Table 3:** Male adolescents: frequency (in %) of suicidal ideation and suicide attempts (lifetime prevalence) and direct self-injurious behavior (12-month prevalence)

N = 10,638		No Migration background	Migration background	Chi^2^	p-value	Bonferroni-Holms corrected sign. Level
Suicidal ideation	No	75.1	74.5	1.339	.720	
	Yes, rarely	17.3	16.9			
	Yes, sometimes	5.5	6.3			
	Yes, often	2.1	2.3			.050
Suicide attempts		3.1	4.7	6.729	**.009**	.013
Direct self-injurious behavior (12-month prevalence)		5.1	6.5	3.892	.049	.017

**Bold:** statistically significant

**Table 4:** Female adolescents: frequency (in %) of suicidal ideation and suicide attempts (lifetime prevalence) and direct self-injurious behavior (12-month prevalence)

N = 10,638		No Migration background	Migration background	Chi^2^	p-value	Bonferroni-Holms corrected sign. Level
Suicidal ideation	No	52.7	51.6	11.738	**.008**	
	Yes, rarely	24.7	23.5			
	Yes, sometimes	13.6	12.8			
	Yes, often	8.9	12.1			.010
Suicide attempts		10.4	15.9	27.972	**<.001**	.006
Deliberate self-harm (12-month prevalence)		29.7	31.8	2.018	.155	.025

The results of the gender-specific analysis concerning suicidal ideation clearly showed that female adolescents (*p* = .008) but not male adolescents (*p* = .720) with a migration background reported a significantly higher frequency of suicidal ideation than female (or male) adolescents without a migration background (Tables 2 and 3). Whereas the descriptive statistics for male adolescents showed an almost equal distribution between the two categories, female adolescents with a migration background reported that they “often” thought about suicide at a rate that was about 1.5 times higher than that of than their native counterparts. In general and independent of migration background, about ¾ of all male adolescents reported that they had never had suicidal thoughts in their lifetime, while only about half of the female sample reported that they had never had suicidal thoughts.

While female adolescents in general reported that they had injured themselves on purpose in the last 12 months at a rate that was about 5 times higher than that of male adolescents (regardless of whether the student had a migration background or not), there were no significant differences between adolescents with and without a migration background. Descriptively, the prevalence was slightly higher for male and female adolescents with a migration background (Tables 3 and 4).

## Discussion

### Comparison of the results with existing epidemiological data

The 12-month prevalence of direct self-injurious behavior in this representative sample of adolescents with a mean age of 15 years was 17.8%. This rate falls in between the rates from German data presented by Plener et al. [2] of 19.8% and Brunner et al. [12] of 14.9%. The rate we identified is close to the pooled rate from 52 international studies of 19.0% for the 12-month prevalence for NSSI [4]. As in many studies reported before, the rate for female adolescents was substantially higher – more than 5 times higher in our study – than for male adolescents [39, 40]. In their meta-analysis of 120 studies, Bresin & Schoenleber [41] concluded that, across age groups, women are more like to engage in NSSI (weighted average Odds Ratio 1.5) and that the effect of sex differences is larger in clinical samples (OR 2.25) than in community samples in adults.

In this study, 15.4% of the adolescents reported that they had engaged in suicidal ideation “sometimes” or “often” in their lifetime. This finding is in line with Brunner et al.’s [12] finding of 14.4% in German adolescents. A total of 36.3% reported that they had ever had suicidal thoughts in their lifetime – about 3% less than reported in the representative German data from 2007 (39.4%) [16]. The finding that about one third of all adolescents had ever had suicidal thoughts in their lifetime corresponds with the range of rates reported by cross-national WHO studies peaking at 37.9% [14]. Just as Kaess et al. [15] showed, we found a clear sex difference with a rate for suicidal ideation that was 2 times higher in female than in male adolescents. Although a recent study in Asia with adolescents and young adults from Malaysia reported a higher rate of suicidal ideation in male individuals [42], there has also been clear support for a higher prevalence in female individuals in other studies [17, 43, 44]. It is possible that the picture in the literature is not clear because there are different sex-specific age peaks in the frequency of suicidal ideation, and thus, results might depend on the mean age of the samples that have been examined. While the prevalence of suicidal ideation appears to peak during mid-adolescence in female adolescents, male adolescents seem to show it in late adolescence instead [45]. Thus, the reason the prevalence rate was higher in female adolescents in our study may have been because the mean age of our sample was 14.9 years.

In our study, 7.6% of the sample reported that they had attempted suicide at least once in their lifetime. This percentage is very close to the results from other representative German studies of adolescents that reported 8.0% [12], 9.0% [16], or 6.5% [2]. The prevalence rate in Germany consistently appears to be lower than the European average of 10.5% [3]. Our result is also comparable to prevalence rates that were based on representative data from the US of adolescents and is also within the reported ranges from pooled international prevalence data [14]. Again, in our data, the prevalence rates for female adolescents were clearly (about 3 times) higher than for male adolescents. A prevalence rate of 2 times higher for female adolescents compared with male adolescents was also reported in another study [15]. In some countries participating in the ESPAD study, female adolescents were again found to attempt suicide at a rate that was 3 times higher than the rate for male adolescents (e.g., in Romania, Greece, or Armenia [3]). The European-wide OSPI project analyzed data on suicide attempts in adults in 8 countries: Gender differences were obvious in the seriousness of the suicide attempt. While actions in men were more often rated as serious suicide attempts, the acts of women were more often categorized as parasuicidal gestures [46]. Thus, having not differentiated the seriousness of the suicide attempts in our study, it could be possible that the prevalence rates we identified for suicide attempts in female adolescents represent a larger share of parasuicidal gestures and thus a more intensive and obvious communication of distress. The difficulties involved in identifying the seriousness of an attempt were underlined by Shaffer [47], who argued that suicide attempts should not be hastily classified as “benign” gestures or “parasuicide.”

### Comparison of the results with data reported by adolescents with a migration background

Our results concerning the higher prevalence rates in the suicide-related variables that we examined are in line with the results presented by Plener et al. [25]. In our sample, adolescents with a migration background had a lifetime prevalence rate of 10.5% for suicide attempts, which was 1.5 times and statistically significantly higher than in German “native” adolescents. The prevalence rates in Plener et al.’s sample of adolescents who had at least one parent who was not born in Germany was 8.94% for suicide attempts with an OR of 4.45 in comparison with German adolescents without a family history of migration. A Swiss study reported that suicide attempts were not related to migration background as the outcome of a multivariate analysis. But in their descriptive data, the rates for lifetime suicide attempts were higher in adolescents with a migration background [26].

The results for direct self-injurious behavior pointed in the same direction. In our sample, adolescents with a migration background reported a 12-month prevalence of 19.6% for having intentionally harmed themselves, while their counterparts without a migration background reported a prevalence of 17.2% (*p* = .006, stat. Sign.). This goes along with Plener at al.’s study on non-suicidal self-injury where significant differences between adolescents with and without a migration background were reported: 30.08% vs. 19.16% lifetime history of NSSI [25].

In our sample, the frequency of suicidal ideation was also higher for adolescents with a migration background, especially concerning the category “often” (7.4% vs. 5.5%; p = .006; stat. Sign.). While the lifetime prevalence of suicidal ideation ranged from 35.9% (natives) to 37.4% (migration background) in our sample, in a Swiss adolescent sample, the 12-month prevalence ranged from 25.9% (natives) to 31.0% (migration background) [26]. However, after controlling for covariates in a regression, migration background lowered the risk for suicidal ideation in the study by Vazsonyi et al. [26].

When interpreting these results, it has to be taken into account that rates of suicide attempts and suicidal ideation vary over time. This point also applies to the prevalence of these constructs in adolescents with a migration background as shown, for example, by Price & Khubchandani [48]. Furthermore it has to be taken into account that suicide rates differ between cultures and religious background which was also reflected in our results depicting suicidal attempts and ideations.

The gender-specific analysis confirmed the general result of a higher prevalence of suicide attempts in (both female and male) adolescents with a migration background in comparison with German “natives.” For suicidal ideation, a statistically significant difference was evident in only the data for female adolescents (*p* = .008) but not for male adolescents with a migration background in comparison with students without a history of migration. The gender-specific analysis did not confirm statistically significant differences in direct self-injurious behavior between adolescents with or without a migration background accounting for both genders.

In looking at adults with a migration background, WHO data have revealed that for many migrant groups, a higher rate of lifetime suicide attempts is reported when compared with adults from the country they immigrated to (“adults without a migration background”). These data also describe an association between the frequency of suicide attempts and migration-specific variables – that is, being born outside the immigration country and retaining citizenship in the “homeland” in later generations (offspring) of migrants, thus pointing to possible obstacles in acculturation [27]. The idea that the suicide attempt of an immigrant is more strongly related to situational stress factors – potentially because of a migrant’s special socio-economic and societal position – and is possibly less persistent over time was pointed out in another study [49]: Even though immigrants are reported to have higher rates of suicide attempts, they are less likely to repeat their suicide attempt in comparison with the “native” population. However, this study again reported on adult data, and these results should not be generalized to adolescents.

Looking at the specifics of the groups with different migration backgrounds, our study revealed the following: Two groups of adolescents with a migration background (i.e., students with roots from Poland and from Southern Europe) showed the highest prevalence rates of deliberate self-harm and suicide attempts. The third highest frequency of suicide attempts was reported by adolescents stemming from predominantly Islamic countries (e.g., Lebanon, Iraq, Iran, Morocco, etc.). It is interesting that this finding is in line with results from Lipsicas et al. [49], who reported that migrant adults from Islamic countries displayed high suicide attempt rates despite low rates in their home countries. As mentioned above, rates vary between countries and one would expect that those variations stay constant during the migration process.

For suicidal ideation, the highest prevalence was found in adolescents with an Asian migration background (49.5%), where almost every second student reported that they had experienced suicidal ideation at least “rarely” in their lifetime. There are hints that Asian adolescents face great challenges in acculturation due to language differences, unfamiliar customs and values [50], and cultural differences in educational practices [51]. One previous study reported that adolescents with an Asian migration background had higher educational aspirations than other immigrant groups [52]. This finding was also supported by our data in that more than 54% of adolescents with Asian migration background aimed for a university entrance diploma (in comparison with 43% of native German students). Such high educational goals are connected with stress itself; additionally experiencing acculturative stress seems to be one argument that can be used to explain the high rate of suicidal ideation in this group. Again, youth with a Polish migration background reported a high lifetime frequency of suicidal ideation (47.5%) followed by adolescents with roots in Northern/Western Europe. In contrast to our study, a report from the Netherlands revealed significantly higher rates in suicidal ideation in adolescents with a Turkish migration background – significantly elevated in comparison with other migrant groups and native adolescents [53]. In our sample, adolescents with a Turkish migration background showed lower rates of suicidal ideation than German natives and several other migrant groups.

When interpreting the results for each of the specific migration groups, it is important to consider that the distribution of gender was not exactly the same in every group. A factor that might partially account for the result for Poland, for example, could be that the number of female adolescents in the sample with a Polish background was 55.9% and thus higher than the average for the total migration sample (49.3%). Since it is known and was also shown in our study that female adolescents showed a higher prevalence of self-harm, suicidal ideation, and suicide attempts, it makes sense for the rates in the Polish-background group to be somewhat higher.

The same explanation can partially account for the relatively low prevalence rates in students with a migration background from the former Yugoslavia; the number of female adolescents in this subsample was 45.0%. However, the proportion of female students does not provide a full explanation for different prevalence rates as demonstrated by the subgroup of adolescents with an Asian background: Despite the fact that the proportion of female students was 45.4% (below average) in this group, the rate of suicidal ideation was clearly elevated in comparison with “German natives.” Also the subsample with a Turkish migration background had a slightly higher proportion of female students (52.4%) than the sample average or the German subsample, and still, the rate of lifetime suicidal ideation and the 12-month prevalence of direct self-injurious behavior were below the average of the total sample of students with a migration background and below the average of the German subsample.

The question of why adolescents with a migration background have a higher prevalence of suicidal ideation and suicide attempts can be discussed with the following hypothesis: Obviously, even second or later generation immigrants suffer from underlying acculturative stress. Lipsicas et al. [49] pointed out that this acculturative stress could be associated with a higher prevalence of suicidal thoughts and behaviors [54]. There is also literature that has shown that especially in adolescents with a migration background, suicidal ideation is associated with life stress and a lack of support from parents [55]. Parents can be a protective factor if they are supportive, if students live with both parents [55], and if parents show an authoritative parenting style [16]. However, migration without parents constitutes a major risk factor [55] – a situation that has become more common under war-related refugee movements in recent years. It seems that the degree of cultural difference between the country or area of origin and the place of resettlement plays an important role in the extent to which “migration background” is a risk factor. When large cultural differences exist, even an internal migration within the same country can be associated with elevated rates of suicidality [56]. Another second hypothesis could be that persons with higher rates of suicidality – be it ideations or attempts – tend to migrate more often. There is a study that showed that suicidal ideation in Mexican adolescents predicted aspirations to migrate to the US [57]. As laid down in the introduction, there are hints in the literature that different variants of acculturation, possibly whether this is in contrast to the acculturation stage of the origin family or to discrimination experiences, might have an association with the frequency of mental symptoms and possibly also to suicidal thoughts and attempts. However, this is up to now speculation and needs to be investigated by analyzing migration specific predictors for suicidal behaviors. This has been done for other risky health behaviours like substance consumption (Donath 2016). However, literature concerning migration-specific predictors on health-related behaviors and more so on mental symptoms is scarce – also because of the necessity of combined medical and sociologic expertise.

A study of adult women with Turkish roots living in Germany assessed the rate of their suicide attempts and their motives for suicidal behavior with a qualitative design. It showed that the dominant motives for suicidal behavior were associated with the individual’s migration history or situation: Young women suffered from a lack of acceptance into German society, and middle-aged women reported isolation and a lack of self-determination [58]. German repatriates emigrating from the former Soviet Union were also previously found to suffer from a lack of acceptance as “Germans” in German society and disillusionment after resettlement; they also showed an elevated risk for suicidality in adulthood [59]. Thus, this would be further hints which underline a possible problematic if there is felt inconsistence between the own felt level of integration or even assimiliation and the view of this status by the others of the majority society. There is a chance that those inconsistence problems are also evident in adolescents and lead to similar risks concerning suicidal thoughts and behavior.

### Limitations

I.) All data analyzed in this study relied on self-reported information. When assessing self-reported data involving personally sensitive information, there is the danger that the respondent will provide socially acceptable answers. This could be a source of bias that can lead to possible underreporting. This would imply that the real prevalence of suicidal behavior and direct self-injurious behavior is higher. However, the prevalence rates reported in the current study are in line with those reported in the literature – also based on self-reports. Thus, we have to assume that the effect size of the bias is about the same in all studies employing the survey method.
II.) The assessment of suicide attempts was carried out with established and tested measurements corresponding to instruments that are widely used on an international level (Self-Harm Behavior Questionnaire [31] and Ottawa Self-Injury Inventory [30]). Yet, the construct of direct self-injurious behavior was assessed without the amendment “without the intention to kill yourself.” In the Ottawa Self-Injury Inventory [30], the wording focuses only on self-injury, without precise actions, but is in line with the focus “without the intention to kill yourself.” To investigate the construct of suicidal ideation, a deviation in scaling and the same time relation as used for the Ottawa Self-Injury Inventory [30] were applied. However, all three constructs were assessed with items that have been established and tested previously in representative surveys [16, 28]. We agree that the choice of instruments is crucial for providing the valid assessment of suicidal ideation and suicide attempts. Published studies have shown high agreement between the existing instruments (Self-Harm Behavior Questionnaire and Ottawa Self-Injury Inventory), which were also used in our study [60].
III.) The method that we used to define migration background differs from that used in other studies, particularly in the US. In our analyses, we did not differentiate between first-generation immigrants and descendants of immigrants living in the immigration country for a second generation. But the latter situation applied to the majority of the current sample. Thus, the present results should be interpreted as accounting for second-generation immigrants. However, the definition used here is the official definition included in the national census surveys and was chosen for comparability with other representative data.
IV.) Since the scope of the study was not suicidality but delinquency and criminological research questions, further clinically interesting data such as history of diagnosed depression or psychiatric/psychotherapeutic interventions because of suicidality were not available.

### Summary and outlook

To our knowledge, this is the first representative study to report data on suicidal ideation, suicide attempts, and direct self-injurious behavior in adolescents with a migration background in Germany. We compared and discussed our results in relation to one non-representative study [25].

The results of our study show that there is a need for the prevention of suicidality in adolescents in Germany and for culturally sensitive prevention in adolescents with a migration background – for female as well as for male adolescents. Target-group-specific prevention should focus on the themes of the target group – for migrants or other groups. There is evidence that such target-specific prevention measures are effective in migrant groups [61]. Further research should explore the specifics of a migration background as a risk factor for suicidal ideation and behavior.

## Conclusions

Our assessment indicates an elevated risk for suicidal behaviors in adolescents with a migration background. This finding is in line with the scarce literature on adolescents with a migration background in Germany on this theme. As others have emphasized before and as suggested by Cramer and Kapusta’s theoretical model [1], the Social-Ecological Framework of Theory, Assessment, and Prevention, there is a need for culturally sensitive prevention that is able to take into account the specific reasons for suicide attempts in migrants.

## Additional file

Additional file 1: Prevalence of suicidal ideation, suicide attempts and direct self-injurious behavior for adolescents who are second generation immigrants versus adolescents without migration background. (DOCX 14 kb)
