# Supplementary material for: Epidemiology of suicidal ideation, suicide attempts, and direct self-injurious behavior in adolescents with a migration background: a representative study
Source: BMC Pediatr. 2019 Feb 1;19:45. doi: 10.1186/s12887-019-1404-z (PMC6359754; doi:10.1186/s12887-019-1404-z)
Supplement: Supplementary file 1 — Prevalence of suicidal ideation, suicide attempts and direct self-injurious behavior for adolescents who are second generation immigrants versus adolescents without migration background. (DOCX 14 kb) [file 12887_2019_1404_MOESM1_ESM.docx]

| **N=10,204** | | **No Migration background (N = 8157)** | **Migration background (2^nd^. generation) (N= 2047)** | **Chi²** | **p-value** |
| --- | --- | --- | --- | --- | --- |
| Suicidal ideation | No | 64.0 | 63.4 | 10.115 | .018 |
| (Life-time prevalence) | Yes, rarely | 21.0 | 19.9 |  |  |
|  | Yes, sometimes | 9.5 | 9.5 |  |  |
|  | Yes, often | 5.5 | 7.3 |  |  |
| Suicide attempts  (Life-time prevalence) | | 6.7 | 9.8 | 22.926 | <.001 |
| Direct self-injurious behavior (12-month prevalence) | | 17.2 | 19.0 | 3.481 | .062 |

Supplementary File 1: Prevalence of suicidal ideation, suicide attempts and direct self-injurious behavior for adolescents who are second generation immigrants versus adolescents without migration background
